# Retinal Microvascular Features Assessed by Optical Coherence Tomography Angiography in Attention Deficit Hyperactivity Disorder

**DOI:** 10.3390/jcm15072669

**Published:** 2026-04-01

**Authors:** Carmen Miquel-Lopez, Jose Javier Garcia-Medina, Antonio Eusebio Lopez-Hernandez, Diego Garcia-Ayuso, Javier Martinez-Soria, Camila Yane-Gauffin, Maria de los Reyes Retamero-Sanchez, Javier Hernandez-Olivares, Monica Del-Rio-Vellosillo

**Affiliations:** 1 General University Hospital Morales Meseguer, 30008 Murcia, Spain; carmenmiquell@gmail.com (C.M.-L.); javiermartiso@hotmail.com (J.M.-S.); camiyanegauffin@hotmail.com (C.Y.-G.); reyesretamero@gmail.com (M.d.l.R.R.-S.); monica.delrio@um.es (M.D.-R.-V.); 2 Multidisciplinary Research Group in Vision and Ophthalmology, University of Murcia, 30003 Murcia, Spain; 3 Department of Ophthalmology, Optometry, Otolaryngology and Pathology, University of Murcia, 30100 Murcia, Spain; antonioeusebio.lopezh@um.es (A.E.L.-H.); diegogarcia@um.es (D.G.-A.); 4 Ophthalmic Research Unit “Santiago Grisolia”, 46010 Valencia, Spain; 5 Spanish Net of Inflammatory Diseases RICORS, Institute of Health Carlos III, 28029 Madrid, Spain; 6 Clinical and Experimental Optometry Research Group, Faculty of Optics and Optometry, University of Murcia, 30003 Murcia, Spain; 7 Experimental Ophthalmology Research Group, Department of Ophthalmology, Optometry, Otolaryngology and Pathology, Faculty of Medicine, University of Murcia, Instituto Murciano de Investigación Biosanitaria Pascual Parrilla—IMIB, 30120 Murcia, Spain; 8 University Hospital Virgen de la Arrixaca, 30120 Murcia, Spain; hdezolivares@gmail.com

**Keywords:** attention deficit hyperactivity disorder, optical coherence tomography angiography, retinal microvasculature, vessel density, perfusion density, superficial capillary plexus, peripapillary microcirculation, biomarker, neurodevelopmental disorders

## Abstract

**Background**: Attention deficit hyperactivity disorder (ADHD) lacks objective biomarkers that may complement clinical diagnosis. Optical coherence tomography angiography (OCTA) enables non-invasive quantitative assessment of retinal microvasculature and has emerged as a potential tool to explore neurovascular features associated with neurodevelopmental disorders. The aim of this study was to comparatively evaluate macular and peripapillary OCTA parameters in individuals with ADHD and neurotypical controls. **Methods**: This comparative case–control study involved 200 eyes (100 from 50 patients with ADHD and 100 from 50 neurotypical controls) belonging to the same well-characterized cohort previously evaluated using structural optical coherence tomography (OCT). Macular and peripapillary OCTA scans were obtained, and quantitative parameters related to vessel density, perfusion density, and peripapillary flow metrics of the superficial retinal capillary plexus were analyzed separately for right and left eyes. Group comparisons were performed using independent-samples *t*-tests and analysis of covariance adjusted for age, sex, and axial length, with correction for multiple comparisons. **Results**: After adjustment for age, sex, and axial length, no OCTA parameter showed statistically significant between-group differences after correction for multiple comparisons. Across parameters, mean differences were small and did not provide statistical evidence of an effect under the prespecified analytical framework. **Conclusions**: Superficial OCTA-derived microvascular parameters did not demonstrate robust between-group differences in ADHD in this cohort. These results suggest that when restricted to the superficial capillary plexus, OCTA metrics are unlikely to serve as standalone biomarkers for ADHD.

## 1. Introduction

Attention deficit hyperactivity disorder (ADHD) is a prevalent neurodevelopmental condition characterized by persistent patterns of inattention, hyperactivity, and impulsivity that interfere with daily functioning [1]. Despite substantial advances in the understanding of its neurobiological underpinnings—including alterations in frontostriatal circuitry and dysregulation of dopaminergic and noradrenergic systems—the diagnosis of ADHD remains primarily clinical. Consequently, there is an increasing interest in identifying objective biomarkers that may complement clinical assessment and contribute to a more comprehensive characterization of the disorder [2].

The retina is embryologically derived from the central nervous system and shares structural, functional, and microvascular characteristics with brain tissue, making it an attractive target for the in vivo study of neurobiological alterations [3]. Optical coherence tomography (OCT) allows high-resolution, non-invasive assessment of retinal microstructure and has been increasingly applied in neurological and psychiatric disorders. Using this approach, we previously demonstrated consistent and bilateral macular retinal thinning in individuals with ADHD, predominantly affecting the total and outer retinal layers, while inner retinal layers and optic nerve head parameters remained largely preserved [4]. These findings support the presence of subtle but measurable retinal structural alterations associated with ADHD. Because neuronal and vascular components of the retina are tightly coupled through neurovascular mechanisms, structural retinal alterations may potentially be accompanied by subtle microvascular adaptations. Therefore, the evaluation of retinal microvasculature using OCT angiography may provide complementary information within a multimodal retinal imaging framework in ADHD.

Optical coherence tomography angiography (OCTA) extends retinal imaging by enabling the non-invasive quantitative assessment of retinal and peripapillary microvasculature without the use of contrast agents. OCTA has shown potential value in a variety of neurological conditions, where microvascular alterations may reflect neurovascular or neurodevelopmental processes [5,6]. In contrast to structural OCT, OCTA primarily provides information on vascular features, potentially capturing complementary aspects of retinal physiology.

In ADHD, however, evidence regarding retinal microvascular changes assessed by OCT angiography remains scarce. To date, only one recent case–control study has compared macular and peripapillary OCT angiography parameters between individuals with attention deficit hyperactivity disorder (ADHD) and neurotypical controls, reporting microvascular differences using analytical approaches without comprehensive adjustment for relevant covariates [7]. Importantly, other quantitative OCTA parameters that may reflect retinal microvascular function, such as perfusion density and flow-related indices, have not yet been systematically explored.

Using the same well-characterized cohort previously evaluated with structural OCT [4], the present study comparatively analyzes macular and peripapillary OCT angiography parameters in individuals with ADHD and neurotypical controls. In addition to conventional vessel density measurements, the analysis incorporates quantitative perfusion density and flow-related metrics, providing a broader characterization of retinal microvascular features. All analyses were performed using covariate-adjusted statistical models with correction for multiple comparisons. This approach aims to clarify the potential contribution of OCTA-derived microvascular metrics as complementary information within a multimodal retinal imaging framework for ADHD.

## 2. Materials and Methods

### 2.1. Study Design and Population

This observational, cross-sectional, comparative case–control study was conducted using the same cohort previously described in our structural optical coherence tomography (OCT) study in attention deficit hyperactivity disorder (ADHD) [4]. The study protocol, ethical approval, informed consent procedures, and demographic characteristics were identical to those previously reported. The study adhered to the tenets of the Declaration of Helsinki and was approved by the Local Ethics Committee of the University of Murcia. Written informed consent was obtained from all participants or their legal guardians.

Participants comprised individuals with a confirmed diagnosis of ADHD and neurotypical control subjects with comparable age and sex distributions. ADHD diagnosis was established according to DSM-5 criteria by experienced clinicians. A detailed description of the inclusion and exclusion criteria has been previously reported [4] and is summarized below for completeness.

### 2.2. Inclusion and Exclusion Criteria

Inclusion criteria for both groups comprised age within the predefined range of the original cohort, best-corrected visual acuity ≥ 20/40, refractive error within ±6.00 diopters spherical equivalent, and ≤2.50 diopters of astigmatism. Exclusion criteria included ocular pathology, amblyopia, congenital ocular abnormalities, history of ocular surgery, systemic diseases potentially affecting retinal structure or microvasculature, and poor-quality OCTA images due to motion artifacts, segmentation errors, or insufficient signal strength.

### 2.3. Ophthalmological Examination

All participants underwent a standardized ophthalmological examination including best-corrected visual acuity, autorefractometry, slit-lamp biomicroscopy, dilated fundus examination, and axial length measurement using a Pentacam AXL system (Oculus Optikgeräte GmbH, Wetzlar, Germany).

### 2.4. OCTA Image Acquisition

OCT angiography (OCTA) images were acquired by the same experienced examiner (C.M.-L.) using the Cirrus HD-OCT 5000 system equipped with AngioPlex software (Carl Zeiss Meditec, Dublin, CA, USA). Macular OCTA scans were obtained using a 6 × 6 mm scan pattern centered on the fovea, while peripapillary OCTA scans covered a 4.5 × 4.5 mm area centered on the optic nerve head.

The AngioPlex platform provides quantitative OCTA measurements exclusively from the superficial retinal capillary plexus, defined by the device as the vascular slab extending from the internal limiting membrane to the inner plexiform layer. Therefore, deeper vascular plexuses were not analyzed in this study. Accordingly, all macular and peripapillary OCTA parameters analyzed in the present study correspond to the superficial capillary plexus.

All OCTA images were reviewed for quality by the same experienced examiner (C.M.-L.), and scans with insufficient signal quality, motion artifacts, decentration, or segmentation errors were excluded from the analysis. Right and left eyes were analyzed separately.

### 2.5. Macular OCTA Parameters

**Figure 1:**
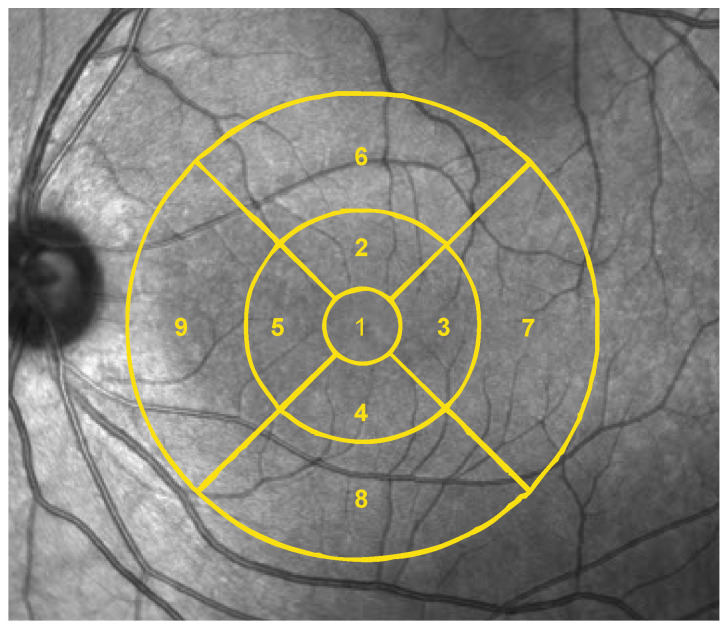
Schematic representation of the ETDRS grid applied to macular OCT angiography analysis of the superficial capillary plexus, illustrating the central, inner, and outer regions used for vessel density and perfusion density assessment. Nine subfields are represented. 1 = center, 2 = inner superior, 3 = inner right, 4 = inner inferior, 5 = inner left, 6 = outer superior, 7 = outer right, 8 = outer inferior, 9 = outer left. The conceptual layout and visualization are adapted from previously published OCTA studies in autism spectrum disorder [8].

Macular OCTA analysis was performed on the superficial capillary plexus using the Early Treatment Diabetic Retinopathy Study (ETDRS) grid, dividing the macula into central, inner, and outer regions (Figure 1).

**Figure 2:**
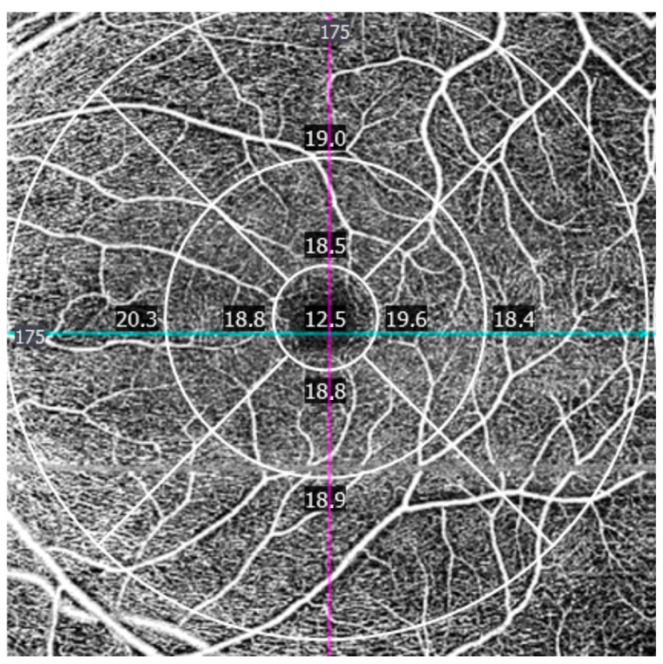
Macular OCT angiography vessel density map of the superficial capillary plexus, illustrating the total length of perfused vasculature per unit area across the macular regions defined by the ETDRS grid. Vessel density values are expressed in mm/mm^2^. The conceptual framework was adapted from prior OCTA studies in autism spectrum disorder [8].

**Figure 3:**
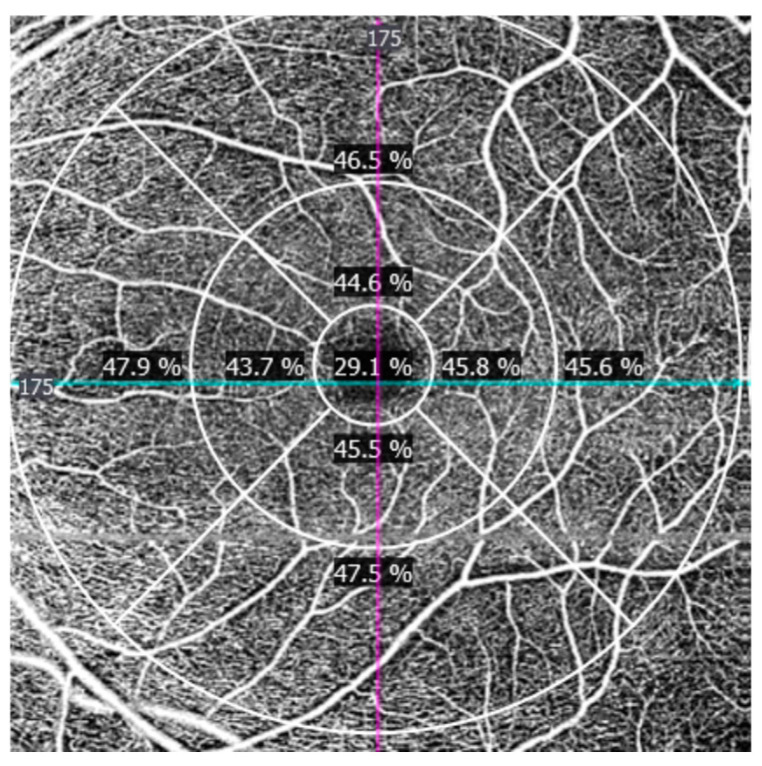
Macular OCT angiography perfusion density map of the superficial capillary plexus, illustrating the proportion of the analyzed area occupied by perfused vasculature across the macular regions. Perfusion density values are expressed as unitless percentages (%). The conceptual framework was adapted from prior OCTA studies in autism spectrum disorder [8].

The following parameters were analyzed:

− Vessel density (VD): defined as the total length of perfused vasculature per unit area, reflecting the spatial extent of the superficial capillary network (Figure 2).

− Perfusion density (PD): defined as the proportion of the analyzed area occupied by perfused vasculature, influenced by vessel caliber (Figure 3).

### 2.6. Peripapillary OCTA Parameters

**Figure 4:**
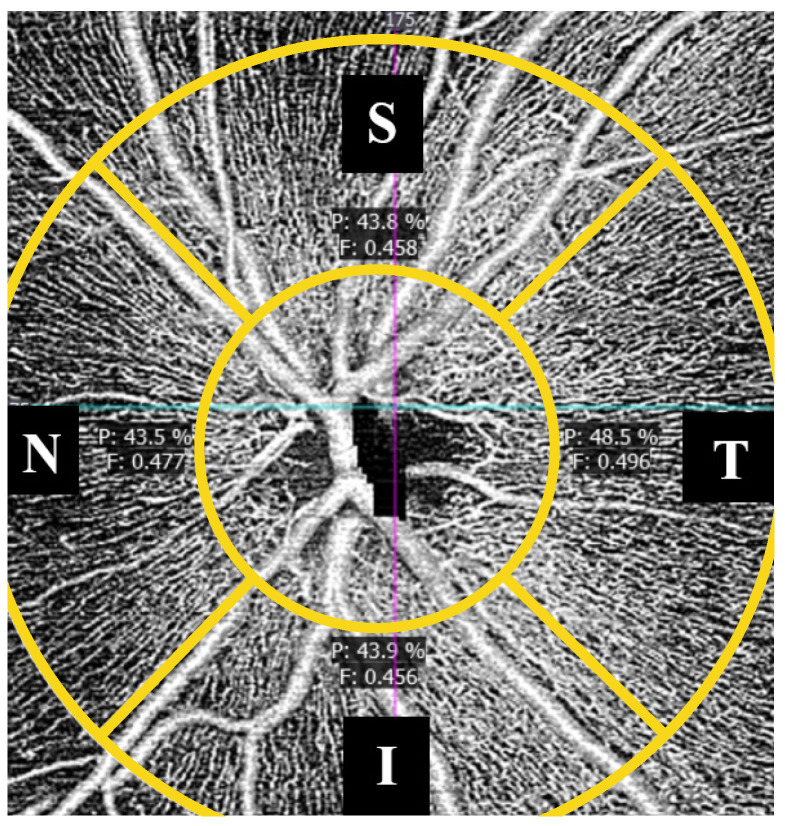
Schematic illustration of peripapillary OCT angiography analysis showing quadrant-based segmentation of the superficial radial peripapillary capillary network surrounding the optic nerve head. Perfusion density (P) is indicated in percentage (%). Flux index (F) is unitless. S = superior, I = inferior, N = nasal, T = temporal. The conceptual framework was adapted from prior OCTA studies in autism spectrum disorder [8].

Peripapillary OCTA analysis evaluated the superficial radial peripapillary capillary network surrounding the optic nerve head. Quantitative measurements were obtained in the superior, inferior, nasal, and temporal quadrants (Figure 4).

The analyzed parameters included:

− Peripapillary perfusion density: defined as the percentage of the peripapillary area occupied by perfused superficial capillaries.
− Flux index: a unitless parameter reflecting the intensity-weighted flow signal within the superficial peripapillary microvasculature, providing an indirect estimate of blood cell flux.

### 2.7. Statistical Analysis

Quantitative variables are expressed as mean ± standard deviation. The distribution of continuous variables was evaluated using the Shapiro–Wilk test. Between-group comparisons of demographic and ophthalmic variables were conducted using independent-samples Student’s *t*-tests, while sex distribution was analyzed using Fisher’s exact test. Between-group comparisons of OCTA parameters were initially conducted using independent-samples Student’s *t*-tests. Subsequently, analysis of covariance (ANCOVA) models adjusted for age, sex, and axial length were applied. Correction for multiple comparisons was performed using the Benjamini–Hochberg false discovery rate procedure. Analyses were performed separately for right and left eyes. Statistical analyses were performed using SPSS (v22.0, SPSS Inc., Chicago, IL, USA) and Python (version 3.11; Python Software Foundation, Wilmington, DE, USA). A *p*-value < 0.05 was considered statistically significant.

## 3. Results

### 3.1. Demographic and Ophthalmic Characteristics

Demographic and baseline ophthalmic characteristics of the study population are summarized in Table 1. As this OCTA analysis was conducted in the same cohort previously reported in our structural OCT study, demographic variables, refractive status, axial length, and keratometric parameters were identical between studies. A total of 112 subjects were initially recruited, of whom 12 were excluded (10 due to poor cooperation and 2 for meeting exclusion criteria). The final sample comprised 100 individuals (200 eyes), including 50 patients with ADHD (34 males and 16 females) and 50 neurotypical controls (25 males and 25 females). No statistically significant differences were observed between groups in terms of age (22.55 ± 5.56 years in controls versus 18.03 ± 6.17 years in the ADHD group; *p* = 0.058), although a trend toward younger age in the ADHD group was noted, or sex distribution (*p* = 0.103). These findings support overall group comparability and, together with the covariate-adjusted analyses, reduce the impact of potential demographic or baseline ophthalmic confounders on subsequent OCTA analyses. Nevertheless, the modest age difference between groups should be acknowledged as a potential source of residual variability in OCTA measurements, given the known sensitivity of retinal vascular metrics to age.

### 3.2. Macular Vessel Density

Macular OCTA parameters related to vessel density of the superficial capillary plexus are presented separately for right and left eyes in Table 2 and Table 3. In unadjusted analyses, several inner macular vessel density sectors showed nominal between-group differences, with values generally tending to be lower in the ADHD group. Importantly, these unadjusted differences showed partial bilateral symmetry across corresponding sectors. After adjustment for age, sex, and axial length using ANCOVA models and correction for multiple comparisons, none of the macular vessel density parameters reached statistical significance in either eye. Effect estimates across most parameters remained small and did not reach statistical significance after adjustment and correction for multiple comparisons, indicating the absence of statistically supported group differences in macular vessel density.

### 3.3. Macular Perfusion Density

Macular perfusion density parameters of the superficial capillary plexus are summarized in Table 4 and Table 5. In the right eye, unadjusted comparisons identified isolated macular regions with nominally lower perfusion density values in ADHD patients compared with controls, with a similar directional pattern observed in the left eye. These unadjusted findings were not robust to covariate adjustment or correction for multiple testing. Following adjustment, no perfusion density parameter remained statistically significant. Despite this, adjusted analyses yielded small effect sizes with consistent directionality across eyes, indicating a pattern of subtle differences that remained within the range of normal inter-individual variability. Importantly, no perfusion density parameter remained statistically significant after covariate adjustment and correction for multiple comparisons.

### 3.4. Peripapillary OCTA Parameters

Peripapillary OCTA parameters, including the perfusion density and flux index of the superficial radial peripapillary capillary network, are presented in Table 6, Table 7, Table 8 and Table 9. In unadjusted analyses, isolated quadrant-level differences were observed, predominantly affecting inferior and superior sectors, with some degree of bilateral correspondence. However, these differences did not persist after adjustment for age, sex, and axial length and correction for multiple comparisons. Across peripapillary parameters, adjusted effect estimates were small and directionally coherent between eyes, supporting the absence of marked peripapillary microvascular alterations while suggesting subtle variations within the limits of physiological variability. Accordingly, none of the evaluated peripapillary OCTA parameters remained statistically significant after multiple-comparison correction.

## 4. Discussion

### 4.1. Principal Findings

In this comparative case–control study using a well-characterized cohort previously examined with structural optical coherence tomography (OCT) [4], we performed a comprehensive evaluation of macular and peripapillary optical coherence tomography angiography (OCTA) parameters derived from the superficial capillary plexus in individuals with attention deficit hyperactivity disorder (ADHD) and neurotypical controls. After adjustment for age, sex, and axial length and correction for multiple comparisons, no OCTA-derived parameter showed statistically significant between-group differences. Nevertheless, several macular and peripapillary metrics exhibited small but directionally consistent differences, often with bilateral symmetry, suggesting subtle group-level trends that did not reach the predefined threshold for statistical significance. Thus, under the prespecified statistical framework, the study yielded negative results with respect to OCTA-derived microvascular parameters. Accordingly, the absence of statistically significant findings after multiple-comparison correction should be interpreted as a lack of evidence for a measurable effect of ADHD on superficial retinal microvasculature under the present analytical conditions.

### 4.2. Group-Level Microvascular Findings in ADHD

The absence of robust adjusted group differences indicates that superficial retinal and peripapillary microvascular architecture is largely preserved in ADHD at the group level. This observation is consistent with findings from OCTA studies in other neurodevelopmental and neuropsychiatric conditions, where microvascular alterations tend to be subtle, heterogeneous, and highly dependent on analytical strategy and covariate adjustment [9,10,11,12]. Large-scale and systematic retinal studies in neurological disease have similarly highlighted substantial inter-individual variability and limited effect sizes when rigorous statistical control is applied [10,11].

### 4.3. Comparison with Previous OCTA Studies in ADHD

In the only previous OCTA case–control study conducted in ADHD, Mahmoud et al. reported microvascular differences primarily affecting vessel density parameters [7]. Notably, the direction of the trends observed in our cohort is consistent with the reductions reported in that study, supporting the biological plausibility of subtle retinal microvascular involvement in ADHD. However, relevant methodological differences—including the lack of comprehensive adjustment for covariates, differences in statistical handling of multiple comparisons, and analytical unit—may partially explain why these directional effects did not translate into statistically significant adjusted group differences in the present analysis.

### 4.4. Statistical Considerations and Interpretation of Directional Trends

In our analysis, several unadjusted comparisons reached nominal significance, particularly within inner macular sectors and selected peripapillary quadrants. Importantly, these findings did not remain significant after covariate adjustment and correction for multiple testing, underscoring the importance of rigorous statistical control when analyzing multiple correlated OCTA parameters [13]. This pattern is well-recognized in OCTA research and highlights the risk of overinterpreting isolated un-adjusted results, particularly in exploratory analyses involving numerous vascular metrics [14]. Importantly, the absence of statistically significant results after correction for multiple comparisons indicates that these directional patterns should not be interpreted as evidence of disease-related microvascular alterations.

Although statistically significant adjusted differences were not identified, effect estimates across most OCTA parameters showed consistent directionality and, in many cases, bilateral correspondence between eyes. Such patterns may reflect subtle microvascular variations that are diluted at the group level due to substantial interindividual variability. Similar observations have been reported in OCTA studies of neurological conditions such as Parkinson’s disease, multiple sclerosis, and mild cognitive impairment, where modest shifts in retinal microvascular metrics coexist with preserved overall vascular architecture [15,16,17].

### 4.5. Relationship with Structural OCT Findings

The present OCTA findings should be interpreted in the context of our previous structural OCT study performed in the same cohort, which demonstrated consistent bilateral macular thinning predominantly affecting the total and outer retinal layers, while inner retinal layers and peripapillary nerve fiber layer thickness were largely preserved [4]. The lack of corresponding alterations in superficial retinal microvasculature is biologically plausible, as the outer retina is primarily supplied by choroidal circulation rather than the superficial retinal capillary plexus assessed by the AngioPlex OCTA platform [18,19]. Comparable dissociations between retinal structural changes and superficial vascular metrics have been described in other neurological disorders [20].

Within the same cohort, previously reported segmented retinal layer thickness measurements obtained by structural OCT showed more consistent group-level alterations than those observed using superficial OCTA metrics [4]. This observation supports the notion that neurodevelopmental changes in ADHD may be more readily captured by structural retinal measurements than by superficial microvascular parameters under cross-sectional analytical conditions.

### 4.6. Pathophysiological Implications

From a pathophysiological perspective, ADHD is increasingly conceptualized as a disorder of neurodevelopmental timing and large-scale network maturation rather than focal neurodegeneration [21,22]. Within this framework, subtle structural retinal alterations may occur without the overt disruption of superficial retinal microvasculature, particularly when assessed cross-sectionally at the group level. This interpretation is consistent with broader dimensional models of neuropsychiatric disorders emphasizing distributed and developmentally mediated alterations rather than focal pathology [23].

### 4.7. Strengths and Limitations

Several strengths enhance the robustness of the present study. First, the use of a well-characterized cohort previously examined with structural OCT enabled a direct comparison between structural and microvascular retinal findings within the same individuals. Second, analyses were performed separately for right and left eyes, allowing the assessment of bilateral consistency. Third, comprehensive covariate adjustment for age, sex, and axial length, together with correction for multiple comparisons, reduced the likelihood of spurious associations. Finally, the inclusion of both macular and peripapillary regions, as well as perfusion- and flow-related metrics, provided a broad characterization of superficial retinal microvasculature. Beyond conventional vessel density measures, the inclusion of perfusion density and flow-related indices may provide additional insight into functional aspects of retinal microcirculation that are potentially more sensitive to subtle neurovascular dysregulation.

Several limitations should also be acknowledged. OCTA measurements were restricted to the superficial retinal capillary plexus, as deeper vascular plexuses and the choriocapillaris cannot be assessed with the AngioPlex platform. The cross-sectional design precludes evaluation of developmental trajectories or longitudinal changes in retinal microvasculature. In addition, inter-individual variability in OCTA parameters may limit the ability to detect small effect sizes at the group level. Finally, the study was designed to address group-level comparisons and was not intended to explore sources of within-group clinical heterogeneity. One additional limitation relates to the absence of widely established pediatric normative OCTA databases. Although this limitation is common in retinal imaging studies involving young populations, its impact on the present analysis was mitigated by the use of an internal neurotypical control group and by covariate-adjusted statistical models enabling robust between-group comparisons. In addition, although right and left eyes were analyzed separately in order to evaluate bilateral consistency of potential OCTA patterns, this approach does not fully model inter-eye correlation at the participant level. Future studies using mixed-effects models or generalized estimating equations may further refine statistical handling of within-subject ocular correlations. Furthermore, although ADHD diagnosis was rigorously established according to DSM-5 criteria, detailed subgroup analyses according to medication status or ADHD subtype were beyond the scope of the present study. This represents a relevant limitation because structural alterations previously identified in this cohort predominantly involved outer retinal layers, which are primarily supplied by the deep capillary plexus and choroidal circulation rather than the superficial plexus evaluated in the present study. In particular, stimulant medications such as methylphenidate may exert systemic or ocular vascular effects that could theoretically influence retinal microvascular measurements. Future studies incorporating medication-stratified analyses and correlations with clinical severity scales (e.g., DSM-5 symptom counts or ADHD-RS scores) may help clarify the clinical relevance of OCTA-derived metrics.

### 4.8. Clinical and Research Implications

From a clinical perspective, the present findings do not support the use of isolated superficial OCTA parameters as standalone biomarkers for ADHD. However, although exploratory patterns were observed, the absence of statistically significant results after multiple-comparison correction indicates that superficial OCTA metrics do not demonstrate consistent microvascular alterations in ADHD. Future studies incorporating deeper vascular plexus analysis, longitudinal designs, and multimodal imaging approaches may further clarify the role of retinal imaging in the study of neurodevelopmental disorders such as ADHD.

## 5. Conclusions

In this well-characterized cohort, superficial retinal microvascular architecture assessed by optical coherence tomography angiography appeared largely preserved in individuals with attention deficit hyperactivity disorder at the group level. No OCTA-derived parameter remained statistically significant after covariate adjustment and correction for multiple comparisons. Small numerical differences were observed across several parameters, but none reached statistical significance after correction for multiple comparisons.

When interpreted alongside previously reported structural OCT findings obtained in the same cohort, these results support the view that retinal alterations associated with ADHD may preferentially manifest at the structural level rather than as overt superficial microvascular changes. Within this framework, OCTA-derived parameters may provide complementary physiological information reflecting subtle group-level tendencies, but do not emerge as robust standalone biomarkers when limited to superficial vascular assessment.

## Figures and Tables

**Table 1:** Comparisons of ophthalmic data between groups for the right and the left eyes. Best-corrected visual acuity (BCVA), spherical equivalent (SE), cycloplegia (cyclo), difference (dif.), attention deficit hyperactivity disorder (ADHD) (adopted from Miquel-Lopez et al., 2025 [4]).

Right Eyes	Control Group	ADHD Group	Mean Dif.	*p* (*t*-Test)
**BCVA**	0.98 ± 0.06	0.95 ± 0.13	0.04	0.533
**SE pre cyclo**	−1.47 ± 2.52	−1.47 ± 2.78	0.01	0.996
**SE post cyclo**	−1.20 ± 2.56	−0.81 ± 2.51	−0.39	0.445
**Axial length**	24.24 ± 1.05	24.12 ± 1.67	0.12	0.104
**K1**	42.08 ± 4.66	42.44 ± 1.57	−0.36	0.602
**K2**	43.68 ± 1.31	43.52 ± 1.71	0.16	0.163
**Left Eyes**	**Control Group**	**ADHD Group**	**Mean Dif.**	***p* (*t*-Test)**
**BCVA**	0.92 ± 0.08	0.96 ± 0.10	−0.04	0.533
**SE pre cyclo**	−1.73 ± 2.46	−1.42 ± 2.97	0.30	0.579
**SE post cyclo**	−1.03 ± 2.47	−0.96 ± 3.05	0.07	0.251
**Axial length**	24.24 ± 1.02	24.12 ± 1.73	0.11	0.683
**K1**	42.65 ± 1.28	42.27 ± 1.75	0.37	0.227
**K2**	43.78 ± 1.40	43.54 ± 1.81	0.23	0.468

**Table 2:** Macular OCTA vessel density values—Right eyes. Values are mean ± SD. Unadjusted comparisons were performed using independent-samples *t*-tests. ANCOVA models were adjusted for age, sex, and axial length (LA). *p*-values were also corrected for multiple comparisons using the Benjamini–Hochberg (BH) false discovery rate procedure. Statistically significant *p*-values (<0.05) are shown in bold.

Parameter	Control Mean ± SD	ADHD Mean ± SD	Mean Diff.	p (*t*-Test)	p (ANCOVA)	p (BH)
**CENTER**	9.183 ± 3.416	8.451 ± 4.095	0.732	0.337	0.184	0.421
**INNER RIGHT**	16.459 ± 2.641	15.228 ± 3.133	1.232	**0.037**	0.089	0.357
**INNER SUPERIOR**	17.040 ± 2.131	16.034 ± 2.543	1.006	**0.036**	**0.040**	0.312
**INNER LEFT**	16.668 ± 2.485	15.948 ± 2.959	0.720	0.194	0.142	0.421
**INNER INFERIOR**	16.936 ± 2.073	15.853 ± 2.862	1.082	**0.034**	**0.043**	0.312
**OUTER RIGHT**	15.485 ± 2.801	14.804 ± 3.210	0.681	0.264	0.479	0.703
**OUTER SUPERIOR**	17.576 ± 1.635	16.916 ± 2.046	0.660	0.080	0.253	0.505
**OUTER LEFT**	19.249 ± 2.140	18.621 ± 2.712	0.629	0.204	0.527	0.703
**OUTER INFERIOR**	17.319 ± 1.796	16.857 ± 2.310	0.461	0.271	0.498	0.703
**CENTRAL MEAN**	9.221 ± 3.476	8.445 ± 4.083	0.775	0.312	0.170	0.421
**INNER MEAN**	16.796 ± 2.107	15.881 ± 2.572	0.914	0.056	0.058	0.312
**OUTER MEAN**	17.136 ± 2.897	16.588 ± 3.037	0.548	0.361	0.635	0.781
**FULL MEAN**	16.767 ± 2.894	16.081 ± 3.029	0.687	0.252	0.438	0.703

**Table 3:** Macular OCTA vessel density values—Left eyes. Values are mean ± SD. Unadjusted comparisons were performed using independent-samples *t*-tests. ANCOVA models were adjusted for age, sex, and axial length (LA). *p*-values were also corrected for multiple comparisons using the Benjamini–Hochberg (BH) false discovery rate procedure. No *p*-values below the 0.05 significance threshold were observed.

Parameter	Control Mean ± SD	ADHD Mean ± SD	Mean Diff.	p (*t*-Test)	p (ANCOVA)	p (BH)
**CENTER**	8.072 ± 3.559	7.359 ± 3.466	0.713	0.313	0.108	0.362
**INNER RIGHT**	15.612 ± 3.192	14.737 ± 3.739	0.875	0.211	0.240	0.481
**INNER SUPERIOR**	16.455 ± 2.354	15.487 ± 3.510	0.969	0.109	0.066	0.362
**INNER LEFT**	15.874 ± 2.754	15.279 ± 3.449	0.595	0.343	0.136	0.362
**INNER INFERIOR**	16.120 ± 2.310	15.380 ± 3.274	0.740	0.195	0.107	0.362
**OUTER RIGHT**	18.817 ± 2.187	18.454 ± 2.375	0.363	0.429	0.587	0.671
**OUTER SUPERIOR**	17.397 ± 1.445	16.645 ± 2.308	0.752	0.054	0.061	0.362
**OUTER LEFT**	15.102 ± 2.781	14.823 ± 2.922	0.279	0.625	0.673	0.718
**OUTER INFERIOR**	17.013 ± 1.882	16.684 ± 2.286	0.330	0.433	0.298	0.530
**CENTRAL MEAN**	8.109 ± 3.601	7.613 ± 3.599	0.496	0.493	0.167	0.383
**INNER MEAN**	15.999 ± 2.415	15.337 ± 3.273	0.661	0.253	0.136	0.362
**OUTER MEAN**	16.886 ± 2.780	16.518 ± 3.114	0.367	0.535	0.571	0.671
**FULL MEAN**	16.383 ± 2.803	15.906 ± 3.201	0.477	0.430	0.428	0.570

**Table 4:** Macular perfusion density—Right eyes. Values are mean ± SD. Unadjusted comparisons were performed using independent-samples *t*-tests. ANCOVA models were adjusted for age, sex, and axial length (LA). *p*-values were also corrected for multiple comparisons using the Benjamini–Hochberg (BH) false discovery rate procedure. Statistically significant *p*-values (<0.05) are shown in bold.

Parameter	Control Mean ± SD	ADHD Mean ± SD	Mean Diff.	p (*t*-Test)	p (ANCOVA)	p (BH)
**CENTER**	0.200 ± 0.079	0.185 ± 0.097	0.015	0.409	0.243	0.359
**INNER RIGHT**	0.381 ± 0.065	0.361 ± 0.074	0.020	0.158	0.202	0.359
**INNER SUPERIOR**	0.407 ± 0.055	0.387 ± 0.060	0.020	0.091	0.068	0.359
**INNER LEFT**	0.389 ± 0.062	0.372 ± 0.072	0.017	0.222	0.155	0.359
**INNER INFERIOR**	0.401 ± 0.052	0.373 ± 0.076	0.028	**0.035**	0.061	0.359
**OUTER RIGHT**	0.372 ± 0.072	0.358 ± 0.083	0.013	0.397	0.566	0.594
**OUTER SUPERIOR**	0.429 ± 0.042	0.419 ± 0.048	0.009	0.298	0.485	0.573
**OUTER LEFT**	0.466 ± 0.057	0.453 ± 0.065	0.013	0.301	0.594	0.594
**OUTER INFERIOR**	0.426 ± 0.051	0.413 ± 0.059	0.013	0.227	0.377	0.491
**CENTRAL MEAN**	0.201 ± 0.081	0.188 ± 0.094	0.013	0.452	0.249	0.359
**INNER MEAN**	0.395 ± 0.053	0.375 ± 0.062	0.020	0.094	0.085	0.359
**OUTER MEAN**	0.424 ± 0.041	0.405 ± 0.056	0.019	0.055	0.203	0.359
**FULL MEAN**	0.411 ± 0.043	0.385 ± 0.067	0.025	**0.028**	0.115	0.359

**Table 5:** Macular perfusion density—Left eyes. Values are mean ± SD. Unadjusted comparisons were performed using independent-samples *t*-tests. ANCOVA models were adjusted for age, sex, and axial length (LA). *p*-values were also corrected for multiple comparisons using the Benjamini–Hochberg (BH) false discovery rate procedure. No *p*-values below the 0.05 significance threshold were observed.

Parameter	Control Mean ± SD	ADHD Mean ± SD	Mean Diff.	p (*t*-Test)	p (ANCOVA)	p (BH)
**CENTER**	0.175 ± 0.080	0.166 ± 0.089	0.010	0.575	0.217	0.314
**INNER RIGHT**	0.360 ± 0.079	0.344 ± 0.089	0.015	0.359	0.322	0.418
**INNER SUPERIOR**	0.390 ± 0.060	0.370 ± 0.083	0.020	0.174	0.071	0.282
**INNER LEFT**	0.367 ± 0.069	0.355 ± 0.084	0.012	0.453	0.150	0.282
**INNER INFERIOR**	0.379 ± 0.061	0.361 ± 0.083	0.018	0.214	0.120	0.282
**OUTER RIGHT**	0.454 ± 0.058	0.444 ± 0.059	0.010	0.398	0.521	0.565
**OUTER SUPERIOR**	0.426 ± 0.040	0.411 ± 0.055	0.015	0.119	0.058	0.282
**OUTER LEFT**	0.361 ± 0.071	0.356 ± 0.073	0.004	0.767	0.719	0.719
**OUTER INFERIOR**	0.414 ± 0.050	0.410 ± 0.061	0.004	0.702	0.440	0.520
**CENTRAL MEAN**	0.176 ± 0.081	0.169 ± 0.083	0.008	0.637	0.212	0.314
**INNER MEAN**	0.374 ± 0.061	0.360 ± 0.079	0.014	0.339	0.152	0.282
**OUTER MEAN**	0.416 ± 0.038	0.399 ± 0.064	0.017	0.116	0.142	0.282
**FULL MEAN**	0.400 ± 0.041	0.383 ± 0.064	0.017	0.118	0.105	0.282

**Table 6:** Peripapillary OCTA perfusion density—Right eyes. Values are mean ± SD. Unadjusted comparisons were performed using independent-samples *t*-tests. ANCOVA models were adjusted for age, sex, and axial length (LA). *p*-values were also corrected for multiple comparisons using the Benjamini–Hochberg (BH) false discovery rate procedure. Statistically significant *p*-values (<0.05) are shown in bold.

Parameter	Control Mean ± SD	ADHD Mean ± SD	Mean Diff.	p (*t*-Test)	p (ANCOVA)	p (BH)
**NASAL**	0.416 ± 0.028	0.422 ± 0.031	−0.006	0.289	0.426	0.533
**SUPERIOR**	0.428 ± 0.021	0.424 ± 0.026	0.004	0.431	0.239	0.533
**TEMPORAL**	0.467 ± 0.027	0.465 ± 0.035	0.002	0.740	0.669	0.669
**INFERIOR**	0.444 ± 0.021	0.432 ± 0.034	0.012	**0.031**	0.182	0.533
**PERFUSION MEAN**	0.438 ± 0.016	0.429 ± 0.041	0.009	0.158	0.339	0.533

**Table 7:** Peripapillary OCTA perfusion density—Left eyes. Values are mean ± SD. Unadjusted comparisons were performed using independent-samples *t*-tests. ANCOVA models were adjusted for age, sex, and axial length (LA). *p*-values were also corrected for multiple comparisons using the Benjamini–Hochberg (BH) false discovery rate procedure. No *p*-values below the 0.05 significance threshold were observed.

Parameter	Control Mean ± SD	ADHD Mean ± SD	Mean Diff.	p (*t*-Test)	p (ANCOVA)	p (BH)
**NASAL**	0.419 ± 0.023	0.411 ± 0.053	0.008	0.359	0.653	0.970
**SUPERIOR**	0.422 ± 0.019	0.424 ± 0.025	−0.002	0.647	0.793	0.970
**TEMPORAL**	0.466 ± 0.026	0.463 ± 0.043	0.003	0.645	0.970	0.970
**INFERIOR**	0.436 ± 0.021	0.426 ± 0.034	0.011	0.060	0.082	0.411
**PERFUSION MEAN**	0.436 ± 0.016	0.428 ± 0.041	0.008	0.215	0.404	0.970

**Table 8:** Peripapillary OCTA flux index—Right eyes. Values are mean ± SD. Unadjusted comparisons were performed using independent-samples *t*-tests. ANCOVA models were adjusted for age, sex, and axial length (LA). *p*-values were also corrected for multiple comparisons using the Benjamini–Hochberg (BH) false discovery rate procedure. Statistically significant *p*-values (<0.05) are shown in bold.

Parameter	Control Mean ± SD	ADHD Mean ± SD	Mean Diff.	p (*t*-Test)	p (ANCOVA)	p (BH)
**NASAL**	0.454 ± 0.036	0.441 ± 0.044	0.013	0.111	0.504	0.647
**SUPERIOR**	0.441 ± 0.028	0.423 ± 0.058	0.018	**0.049**	0.288	0.647
**TEMPORAL**	0.476 ± 0.040	0.459 ± 0.051	0.017	0.075	0.467	0.647
**INFERIOR**	0.444 ± 0.027	0.438 ± 0.032	0.006	0.337	0.746	0.746
**FLUX MEAN**	0.455 ± 0.031	0.442 ± 0.047	0.013	0.105	0.539	0.647

**Table 9:** Peripapillary OCTA flux index—Left eyes. Values are mean ± SD. Unadjusted comparisons were performed using independent-samples *t*-tests. ANCOVA models were adjusted for age, sex, and axial length (LA). *p*-values were also corrected for multiple comparisons using the Benjamini–Hochberg (BH) false discovery rate procedure. Statistically significant *p*-values (<0.05) are shown in bold.

Parameter	Control Mean ± SD	ADHD Mean ± SD	Mean Diff.	p (*t*-Test)	p (ANCOVA)	p (BH)
**NASAL**	0.440 ± 0.044	0.424 ± 0.048	0.016	0.093	0.304	0.365
**SUPERIOR**	0.438 ± 0.031	0.421 ± 0.041	0.017	**0.024**	0.067	0.176
**TEMPORAL**	0.473 ± 0.039	0.449 ± 0.055	0.024	**0.014**	0.051	0.176
**INFERIOR**	0.438 ± 0.032	0.424 ± 0.036	0.014	**0.041**	0.117	0.176
**FLUX MEAN**	0.448 ± 0.036	0.430 ± 0.048	0.018	**0.032**	0.100	0.176

## Data Availability

The datasets generated and/or analyzed during this study are available from the corresponding author on reasonable request.

## References

[1] American Psychiatric Association (2013). Diagnostic and Statistical Manual of Mental Disorders.

[2] Faraone S.V., Bellgrove M.A., Brikell I., Hartman C.A., Cortese S., Newcorn J.H., Hollis C., Polanczyk G.V., Philipson A., Rubia K. (2024). Attention-Deficit/Hyperactivity Disorder. Nat. Rev. Dis. Primers.

[3] London A., Benhar I., Schwartz M. (2013). The retina as a window to the brain—From eye research to CNS disorders. Nat. Rev. Neurol..

[4] Miquel-López C., García-Medina J.J., López-Hernández A.E., García-Ayuso D., De-Paco-Matallana M., Hernández-Olivares J., Pinazo-Durán M.D., Del-Río-Vellosillo M. (2025). Retinal Thinning in Attention Deficit Hyperactivity Disorder (ADHD): Structural Changes Detected by Spectral-Domain OCT. J. Clin. Med..

[5] Ong Y.T., Hilal S., Cheung C.Y., Xu X., Chen C., Venketasubramanian N., Wong T.Y., Ikram M.K. (2014). Retinal Vascular Fractals and Cognitive Impairment. Dement. Geriatr. Cogn. Dis. Extra.

[6] Augustin A.J., Atorf J. (2022). The Value of Optical Coherence Tomography Angiography (OCT-A) in Neurological Diseases. Diagnostics.

[7] Mahmoud H., Korishy M.S.H., Amin A.E., Awny I., Tohamy D. (2025). Optical Coherence Tomography Angiography Findings in Attention-Deficit/Hyperactivity Disorder. BMC Ophthalmol..

[8] García-Medina J.J., Rubio-Velázquez E., López-Bernal M.D., Párraga-Muñoz D., Pérez-Martínez A., Pinazo-Durán M.D., Del-Río-Vellosillo M. (2020). Optical Coherence Tomography Angiography of Macula and Optic Nerve in Autism Spectrum Disorder: A Pilot Study. J. Clin. Med..

[9] Lahme L., Esser E.L., Mihailovic N., Schubert F., Lauermann J., Johnen A., Eter N., Duning T., Alnawaiseh M. (2018). Evaluation of Ocular Perfusion in Alzheimer’s Disease Using Optical Coherence Tomography Angiography. J. Alzheimer’s Dis..

[10] Den Haan J., Verbraak F.D., Visser P.J., Bouwman F.H. (2017). Retinal Thickness in Alzheimer’s Disease: A Systematic Review and Meta-Analysis. Alzheimer’s Dement..

[11] Mutlu U., Cremers L.G., de Groot M., Hofman A., Niessen W.J., van der Lugt A., Klaver C.C.W., Ikram M.A., Vernooij M.W., Ikram M.K. (2016). Retinal Microvasculature and White Matter Microstructure: The Rotterdam Study. Neurology.

[12] Kennedy K.G., Mio M., Goldstein B.I., Brambilla P., Delvecchio G. (2023). Systematic Review and Meta-Analysis of Retinal Microvascular Caliber in Bipolar Disorder, Major Depressive Disorder, and Schizophrenia. J. Affect. Disord..

[13] Benjamini Y., Hochberg Y. (1995). Controlling the false discovery rate: A practical and powerful approach to multiple testing. J. R. Stat. Soc. Ser. B.

[14] Spaide R.F., Fujimoto J.G., Waheed N.K., Sadda S.R., Staurenghi G. (2018). Optical Coherence Tomography Angiography. Prog. Retin. Eye Res..

[15] Robbins C.B., Thompson A.C., Bhullar P.K., Koo H.Y., Agrawal R., Soundararajan S., Yoon S.P., Polascik B.W., Scott B.L., Grewal D.S. (2021). Characterization of Retinal Microvascular and Choroidal Structural Changes in Parkinson Disease. JAMA Ophthalmol..

[16] Gernert J.A., Zausinger H., Diedrich L., Wicklein R., Kreitner L., Kümpfel T., Havla J. (2026). The Potential of Optical Coherence Tomography Angiography in Progressive Multiple Sclerosis. J. Neurol..

[17] Jiang H., Wei Y., Shi Y., Wright C.B., Sun X., Gregori G., Zheng F., Vanner E.A., Lam B.L., Rundek T. (2018). Altered Macular Microvasculature in Mild Cognitive Impairment and Alzheimer Disease. J. Neuroophthalmol..

[18] Nickla D.L., Wallman J. (2010). The multifunctional choroid. Prog. Retin. Eye Res..

[19] Pournaras C.J., Rungger-Brändle E., Riva C.E., Hardarson S.H., Stefansson E. (2008). Regulation of Retinal Blood Flow in Health and Disease. Prog. Retin. Eye Res..

[20] Cordon B., Vilades E., Orduna E., Satue M., Perez-Velilla J., Sebastian B., Polo V., Larrosa J.M., Pablo L.E., Garcia-Martin E. (2020). Angiography with Optical Coherence Tomography as a Biomarker in Multiple Sclerosis. PLoS ONE.

[21] Shaw P., Eckstrand K., Sharp W., Blumenthal J., Lerch J.P., Greenstein D., Clasen L., Evans A., Giedd J., Rapoport J.L. (2007). Attention-deficit/hyperactivity disorder is characterized by a delay in cortical maturation. Proc. Natl. Acad. Sci. USA.

[22] Castellanos F.X., Proal E. (2012). Large-scale brain systems in ADHD: Beyond the prefrontal–striatal model. Trends Cogn. Sci..

[23] Insel T., Cuthbert B., Garvey M., Heinssen R., Pine D.S., Quinn K., Sanislow C., Wang P. (2010). Research Domain Criteria (RDoC): Toward a New Classification Framework for Research on Mental Disorders. Am. J. Psychiatry.

